# Parents’ experience in the Ronald McDonald Houses

**DOI:** 10.1192/j.eurpsy.2022.2242

**Published:** 2022-09-01

**Authors:** M. Cosquer, C. Jousselme

**Affiliations:** Fondation Vallée Hospital, Research Department, Gentilly, France

**Keywords:** accomodation, child hospitalization, family centered care, family experience

## Abstract

**Introduction:**

Child hospitalization is a difficult event in the life for the whole family, probably worst for families coming from far away, specially for accommodation. Ronald McDonald Houses (RMH) created in the immediate proximity of pediatric departments of hospitals, allows hospitalized children to benefit from the presence of his family nearby. The hypothesis that the family is stronger when it is grouped together (the “family-centered-care” concept) remains difficult to demonstrate (Cochrane, 2012). In France, there is no study describing the interest of such places, and families experiences.

**Objectives:**

Our objectives are to describe the experience of parents in French RMH.

**Methods:**

A cross-sectional study conducted between February and April 2016, invited 50 families to participate in the 9 French RMH, by feeling an anonymous questionnaire (socio-demographic characteristics, items related to hospitalization, anxiety and depression scale -HADS, conditions of stay at the house). Descriptive statistics presented mothers and fathers experience.

**Results:**

Parents of 333 hospitalized child participated : 320 mothers, 246 fathers. 44.1% of child were aged less than one year. Services more represented were : intensive care unit, oncology and neonatal. Parents were socially rather disadvantaged, living mainly in couples, with an estimated mean home-hospital time of 2 hours. They reported financial problems (>40%), sleep deprivation (>1.5 hours), and anxiety-depressive disorders: anxiety (>50%) and depression (>20%). Satisfaction staying in the house was extremely high (>95%).

**Conclusions:**

We observe an undeniable added value of the RMH in the care. Nevertheless, the high level of psychological suffering shows the importance of offering help at the psycho-social level.

**Disclosure:**

No significant relationships.

